# Triglycerides Promote Lipid Homeostasis during Hypoxic Stress by Balancing Fatty Acid Saturation

**DOI:** 10.1016/j.celrep.2018.08.015

**Published:** 2018-09-04

**Authors:** Daniel Ackerman, Sergey Tumanov, Bo Qiu, Evdokia Michalopoulou, Michelle Spata, Andrew Azzam, Hong Xie, M. Celeste Simon, Jurre J. Kamphorst

**Affiliations:** 1 Abramson Family Cancer Research Institute, University of Pennsylvania Perelman School of Medicine, Philadelphia, PA 19104, USA; 2 Cancer Research UK Beatson Institute, Garscube Estate, Switchback Road, Glasgow G61 1BD, UK; 3 Institute of Cancer Sciences, University of Glasgow, Garscube Estate, Switchback Road, Glasgow G61 1QH, UK

**Keywords:** cancer metabolism, clear cell renal cell carcinoma, diglyceride acyltransferase, fatty acid saturation, hypoxia, lipid droplets, lipid homeostasis, lipidomics, stable isotope tracing, triglycerides

## Abstract

Lipid droplets, which store triglycerides and cholesterol esters, are a prominent feature of clear cell renal cell carcinoma (ccRCC). Although their presence in ccRCC is critical for sustained tumorigenesis, their contribution to lipid homeostasis and tumor cell viability is incompletely understood. Here we show that disrupting triglyceride synthesis compromises the growth of both ccRCC tumors and ccRCC cells exposed to tumor-like conditions. Functionally, hypoxia leads to increased fatty acid saturation through inhibition of the oxygen-dependent stearoyl-CoA desaturase (SCD) enzyme. Triglycerides counter a toxic buildup of saturated lipids, primarily by releasing the unsaturated fatty acid oleate (the principal product of SCD activity) from lipid droplets into phospholipid pools. Disrupting this process derails lipid homeostasis, causing overproduction of toxic saturated ceramides and acyl-carnitines as well as activation of the NF-κB transcription factor. Our work demonstrates that triglycerides promote homeostasis by “buffering” specific fatty acids.

## Highlights

• Hypoxia influences the FA composition of TGs more than other lipid classes
• TGs maintain the cell’s FA saturation index by active oleate exchange
• Release of TG-resident oleate during hypoxia prevents production of toxic lipids
• Disrupting TG synthesis compromises tumor growth

## Graphical Abstract

**Figure:**
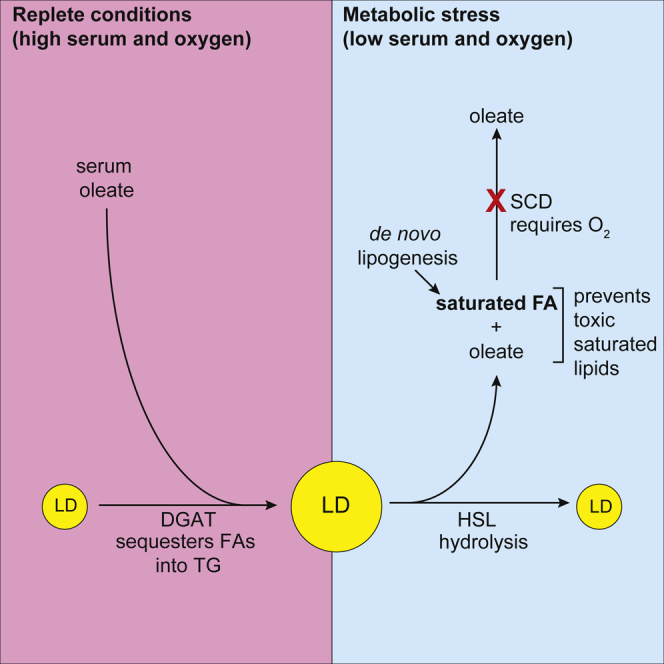

## Teaser

Tumors frequently experience hypoxia and serum limitation, which cause a harmful increase in fatty acid saturation. Studying kidney cancer, Ackerman et al. describe a protective role of lipid droplet-resident triglycerides: buffering of the cellular lipid saturation through exchange of mono-unsaturated fatty acids. Inhibiting triglyceride synthesis compromises solid tumor growth.

## Introduction

**Figure 1:**
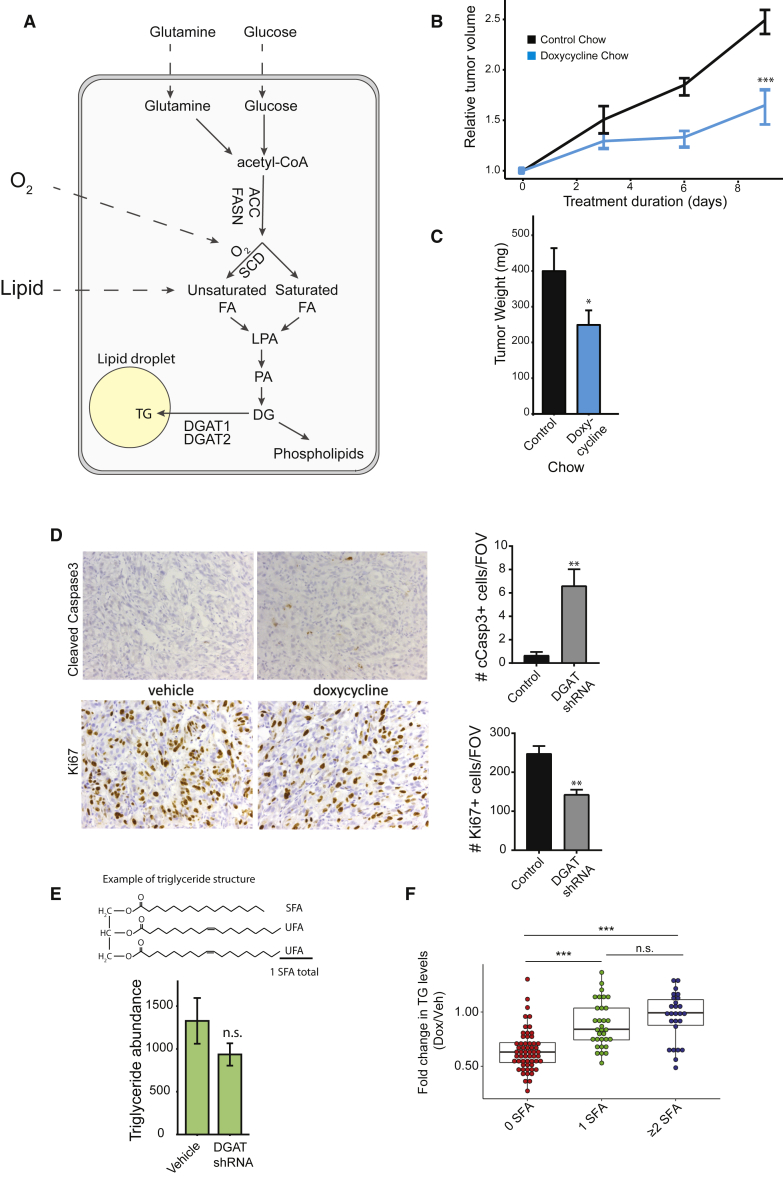
DGAT Loss Reduces Tumor Growth and Alters Lipid Composition *In Vivo* (A) Diagram of fatty acid and lipid synthesis and the influence of O_2_ and exogenous lipid. (B) Growth curves for A498 xenograft tumors with induced (doxycycline chow) and un-induced (control chow) *DGAT1* and *DGAT2* shRNAs (hereafter called *DGAT* shRNA). (C) Tumor weights after necropsy. (D) Immunohistochemistry for cleaved caspase-3 and Ki67 in xenograft tumors collected on day 5 of treatment, with accompanying quantification. (E) Total TG abundance derived from summing individual TG species abundance after liquid chromatography-mass spectrometry (LC-MS) quantification. (F) TG species binned according to the number of fully saturated FA chains present and the abundance of each category summed and displayed as a ratio of doxycycline-treated versus control groups. All results are means of n = 10 tumors (2 tumors per mouse) per arm; error bars represent ± SD (B, D, and F) or ± SEM (C). Statistical significance by t test or ANOVA, as appropriate; ^∗^p < 0.05, ^∗∗^p < 0.01, and ^∗∗∗^p < 0.001. ACC, acetyl-CoA carboxylase; CE, cholesterol ester; DG, diglyceride; DGAT, diglyceride acyltransferase; FASN, fatty acid synthase; ns, non-significant; PC, phosphatidylcholine; PE, phosphatidylethanolamine; PS, phosphatidylserine; SCD(i), stearoyl-CoA desaturase (inhibitor); SFA, saturated FA; TG, triglyceride; UFA, unsaturated FA. See also Figure S1.

Proliferating cancer cells exhibit an increased dependence on biosynthetic intermediates (Vander Heiden and DeBerardinis, 2017), including fatty acids (FAs) that support the construction of organelle and plasma membranes. To meet the demand for elevated FA levels, FA synthase (FASN) overexpression is commonly observed in multiple cancers (Menendez and Lupu, 2007, Ricoult et al., 2016). Palmitate, the product of FASN enzymatic activity, can be further modified by elongation and desaturation, where double bonds between carbon atoms are introduced into long-chain FAs. Stearoyl-coenzyme A (CoA) desaturase (SCD), the principal enzyme responsible for desaturation, is critical for sustained viability of a variety of tumor cell types (Igal, 2016). By introducing a double bond into the saturated FA stearate, SCD produces monounsaturated oleate, typically the most abundant intracellular FA. Although clearly important for cell survival, activity of the oxygen (O_2_)-dependent SCD enzyme can be constrained by tumor hypoxia (Figure 1A). Periods of O_2_ starvation, therefore, lead to a buildup of saturated FA precursors, causing disruption of endoplasmic reticulum (ER) membranes and apoptosis (Kamphorst et al., 2013, Young et al., 2013). Saturated FA-induced toxicity can be alleviated by supplying exogenous unsaturated lipids (for instance, by increasing the availability of serum FAs), indicating that lipid uptake is an important mechanism for maintaining homeostasis in hypoxic cancer cells (Young et al., 2013).

Accentuated accumulation of neutral lipids in large lipid droplets (LDs) is observed in a subset of tumor types, particularly clear cell renal cell carcinoma (ccRCC). In ccRCC, this phenotype has been linked to genetic loss of the von Hippel-Lindau (*VHL*) tumor suppressor, which causes constitutive hypoxia inducible factor-α (HIFα) stabilization regardless of O_2_ availability. We have previously shown that induction of HIF2α specifically promotes lipid accumulation through upregulation of *PLIN2*, the gene encoding the LD coat protein perilipin-2 (Qiu et al., 2015). PLIN2 loss significantly represses tumor growth, indicating that LD formation may be driven by HIF2α stabilization and serves a cytoprotective role in ccRCC. In a separate study, HIF-dependent repression of FA β-oxidation has also been demonstrated to contribute to LD accumulation (Du et al., 2017).

LDs are primarily composed of cholesterol esters (CEs) and triglycerides (TGs), and lipidomic analyses of ccRCC samples revealed high levels of both in tumors compared with normal kidney (Saito et al., 2016, Sundelin et al., 2012). TGs consist of a glycerol backbone and three FAs (Figure 1E), with a significant diversity in FA chain length and number of double bonds. Their synthesis requires the activity of the diglyceride acyltransferase (DGAT) enzymes DGAT1 and DGAT2, which catalyze the condensation of fatty acyl-CoA with a diglyceride (DG) to form TG. The two human DGAT enzymes share no homology and have dissimilar expression patterns (Yen et al., 2008). TGs are synthesized in the ER, but DGAT2 can also be found on the surface of LDs and may generate TGs in growing LDs *in situ* (Wilfling et al., 2013). To mobilize lipid stores to provide FAs, TGs are broken down by a series of lipases, and the released FAs can, in principle, be used for incorporation into other lipid types, such as phospholipids (PLs), or for mitochondrial oxidation. Although the protective function of LDs and TG turnover have been identified in a number of contexts (Bailey et al., 2015, Bensaad et al., 2014), the full scope of TG synthesis and catabolism in tumor cells remains unclear.

Here we evaluated the consequences of limiting TG synthesis in ccRCC. We found that concurrent inhibition of DGAT1 and DGAT2 severely compromised *in vivo* tumor growth because of increased cell death. This was replicated in cultured cells exposed to low O_2_ and serum, mimicking a stressful tumor microenvironment. Mechanistically, TGs sequester exogenous unsaturated FAs, particularly oleate, when in ample supply. When oleate availability becomes limiting during O_2_ and serum deprivation, however, oleate is instead released into other lipid pools. This prevents the buildup of fully saturated, toxic lipids in cellular compartments outside of LDs. Our work reveals a dynamic mechanism by which TGs act as buffers for cellular lipid homeostasis, especially under the tumor-relevant conditions of O_2_ and nutrient limitation.

## Results and Discussion

### Disruption of TG Synthesis Compromises ccRCC Tumor Growth

Although the functional roles of CEs in cancer have been interrogated to some extent (Yue et al., 2014), TGs have so far remained considerably less well studied. We investigated how direct disruption of TG synthesis by loss of DGAT enzymes affects lipid homeostasis. DGATs appear to carry out mutually redundant functions in the storage of both endogenously synthesized and exogenously derived FAs (Figure 1A). We confirmed their redundancy in A498 ccRCC cells by examining the induction of neutral lipid stores upon administration of oleic acid conjugated with BSA versus BSA alone. Although a combination of CRISPR/Cas9-mediated *DGAT2* deletion and DGAT1 pharmacological inhibition fully abrogated this, loss of neither DGAT individually was sufficient (Figure S1A). This approach provides the opportunity to precisely control the timing of DGAT inhibition by adding the DGAT1 inhibitor T863 (DGAT1i) to cells with *DGAT2* deletion. Importantly, *DGAT* deficiency was complemented with a CRISPR-resistant *DGAT2* cDNA, restoring neutral lipid deposition (Figure S1A). To study the consequences of TG synthesis inhibition *in vivo* (and employ a complementary approach), we generated A498 cells expressing both *DGAT1* and *DGAT2* short hairpin RNAs (shRNAs) under the control of a Tet-inducible promoter and confirmed that these constructs effectively reduce *DGAT* mRNA and protein levels upon doxycycline treatment (Figures S1B and S1C). After implanting these cells subcutaneously in immunocompromised recipients and allowing tumors to grow to an average size of 300 mm^3^, mice were fed with either control or doxycycline-containing chow. A substantial reduction in both *DGAT1* and *DGAT2* transcript levels (Figure S1D) and significantly reduced tumor volume and weight was observed (Figures 1B and 1C). Immunohistochemical staining of tumor sections revealed increased numbers of apoptotic cells based on cleaved caspase-3 staining and decreased numbers of actively dividing Ki67+ cells (Figure 1D). As anticipated, TG levels were lower in DGAT-deficient tumors compared with controls (Figure 1E; Table S1), although the observed differences failed to reach statistical significance. These results likely reflect the inherent heterogeneity between cells within solid tumors with regard to TG synthesis and turnover, in addition to variable O_2_ and nutrient availability. Nevertheless, a pronounced increase in TGs containing one or more saturated FAs (≥1 saturated FAs [SFAs]; Figure 1F), but not those exclusively carrying unsaturated FAs (0 SFAs; Figure 1F) was observed. Thus, *DGAT* silencing disrupts TG FA composition and causes both increased apoptosis and reduced proliferation of ccRCC tumor cells *in vivo*.

### DGAT Loss Compromises ccRCC Viability in Low O_2_ and Serum

**Figure 2:**
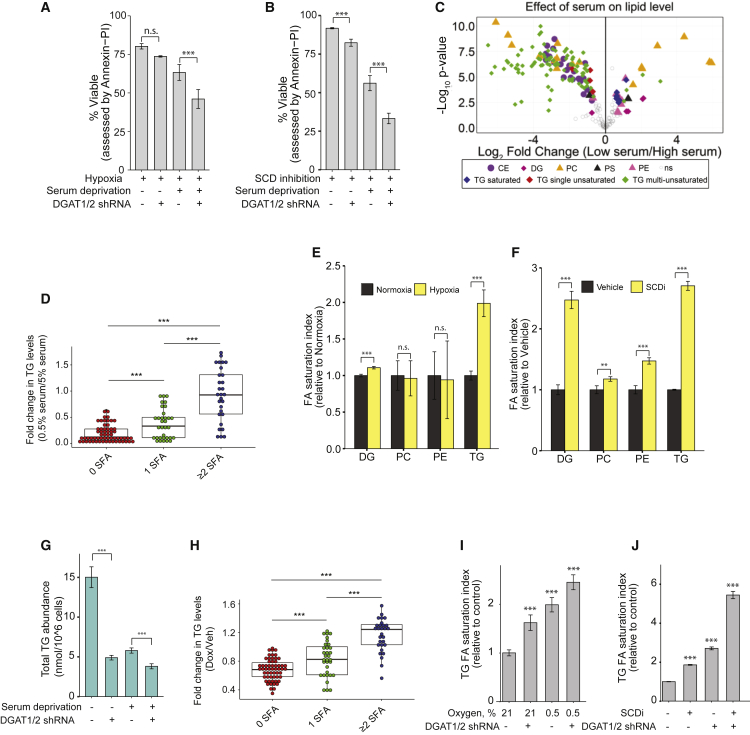
TGs Promote Cell Viability in Low O_2_ and Serum by Absorbing FA Saturation (A) Viability of A498 cells expressing inducible shRNA against *DGAT1* and *DGAT2* mRNAs (*DGAT* shRNA), assessed after 72 hr under the indicated conditions (hypoxia = 0.5% O_2_; serum deprivation = low serum, 0.5% fetal bovine serum [FBS]) by Annexin-propidium iodide (PI) flow cytometry assay. (B) Viability of cells expressing inducible *DGAT* shRNAs after 72 hr under the indicated conditions (SCDi, 1 μM CAY10566) by Annexin-PI assay using flow cytometry. (C) Volcano plot showing fold change and significance of alterations in the lipidome of A498 cells cultured in low (0.5%) versus high (5%) serum. Lipids with ≥ 1.5 fold change and p ≤ 0.05 are displayed in color to denote lipid class. (D) Changes in FA composition or saturation of TGs, calculated by aggregating TG abundances for species containing 0, 1, or 2+ SFA chains separately. Values are normalized to control conditions (5% serum). (E) Lipid class-specific saturation indices (defined by (palmitate + stearate) / oleate) for A498 cells cultured under hypoxic (0.5% O_2_) versus normoxic conditions (both in low serum). (F) As (E) but with pharmacological SCD inhibition (1 μM CAY10566) instead of hypoxia. (G) Effect of serum deprivation and *DGAT* shRNA on total TG abundances. (H) Changes in FA makeup of TGs following DGAT knockdown; values were calculated by aggregating TG abundances for species containing 0, 1, or 2+ SFA chains separately. Values were normalized to the control condition (vehicle [Veh] treatment). (I) TG saturation indices for the indicated conditions. Values are relative to normoxic untreated cells. (J) As (G) but with pharmacological SCD inhibition (1 μM CAY10566). Values are relative to the untreated vehicle control. Data are means of 3 (A, B, and D–J) or 5 (C) replicate wells and were confirmed in independent experiments; error bars represent SD. Statistical significance by t test or ANOVA, as appropriate. ^∗∗^p < 0.05, and ^∗∗∗^p < 0.005. See also Figure S2.

We next sought to establish exactly how TG metabolism promotes tumor growth. Because solid tumors are notoriously poorly perfused and hypoxic (Frantz et al., 2010), we specifically focused on how serum lipid and O_2_ limitation results in DGAT dependency. Of note, combined serum and O_2_ limitation led to deteriorated cell viability upon DGAT knockdown (Figure 2A). Because hypoxia limits SCD activity (Figure S2A) and reduces cell viability in the absence of exogenous lipid supply (Kamphorst et al., 2013, Qiu et al., 2015), we investigated whether enhanced sensitivity of DGAT-deficient cells to these conditions is indeed mediated by reduced SCD function. Cells were exposed to the SCD inhibitor CAY10566, which phenocopied the effect of O_2_ deprivation (Figure 2B).

To understand the relationship between serum levels and TG metabolism, a lipidomic comparison of A498 cells cultured in either high (5%) or low (0.5%) serum-containing medium was performed, revealing substantial remodeling of the intracellular lipid composition. Among the most pronounced changes were significant reductions in CEs as well as in TGs (Figure 2C). Limiting serum *in vitro* led to large decreases in the abundance of unsaturated TGs (Figure 2C) and a shift toward TG saturation (Figure 2D), as noted for solid tumors (Figures 1E and 1F). A striking depletion in neutral lipid stores was also confirmed by *boron-dipyrromethene* (BODIPY) imaging (Figure S2B), in line with earlier observations (Bensaad et al., 2014). This indicates that, in addition to HIF signaling, availability of exogenous serum lipids is critical for maintaining abundant lipid stores. Increased TG storage observed under hypoxia appears to be cytoprotective and renders cells more resistant to subsequent hypoxia and hypoxia-reoxygenation-mediated cytotoxicity (Bensaad et al., 2014). Moreover, TGs may also harbor polyunsaturated FAs to protect them against peroxidation (Bailey et al., 2015). To establish whether lipid stores “buffer” against lipid saturation in our system, we determined whether any one class of lipid preferentially “absorbs” alterations in FA saturation under hypoxia. Strikingly, TG composition was affected much more profoundly than other lipid classes (Figures 2E and S2C), including a loss of TGs harboring unsaturated FAs and a shift toward increased TG saturation (Figure S2D). Moreover, these changes were only observed under low-serum conditions. When A498 cells were exposed to pharmacological SCD inhibition instead of hypoxia (Figure S2E), we observed a similar but more pronounced increase in TG saturation (Figure 2F) as well as a stronger effect on other lipid classes (especially DGs), the direct precursors for TGs. This difference is most likely due to a combination of more potent SCD inhibition by pharmacological approaches compared with hypoxia (leading to increased FA saturation) as well as a limited capacity of TGs to cope with the increased FA saturation, leading to “spill-over” into other lipid classes. Importantly, when other ccRCC cell lines (786-O and UMRC2) were exposed to low O_2_, FA saturation was similarly most pronounced in TGs, followed by the direct precursor DGs (Figures S2F and S2G). This suggests that TGs have a capacity to promote cell viability by balancing the availability of specific FAs.

Serum deprivation reduced intracellular TG abundance (Figure 2G), in keeping with the BODIPY imaging depicted in Figure S2B. As demonstrated with *in vivo* tumor growth (Figures 1E and 1F), *DGAT* silencing *in vitro* also caused a further decrease in TG abundance (Figure 2G) and selective depletion of unsaturated TGs (Figure 2H). Given that both hypoxia and DGAT depletion cause increased saturation of the TG pool under low-serum conditions, we asked whether combination of the two leads to a cumulative effect; i.e., an even more saturated TG pool. Indeed, in the setting of low serum availability, O_2_ deprivation or pharmacological SCD inhibition increased TG saturation (Figures 2I and 2J). Again, more potent pharmacological SCD inhibition (compared with hypoxia) resulted in increased TG saturation relative to O_2_ starvation. Additional *DGAT* knockdown further lowered TG levels (Figure 2G), resulting in an even more saturated TG pool (Figures 2I and 2J). These results highlight a protective role of LDs under serum- and O_2_-limited conditions through buffering cellular FA saturation.

### TGs Neutralize Excess Fatty Acid Saturation through Release of Stored Oleate

**Figure 3:**
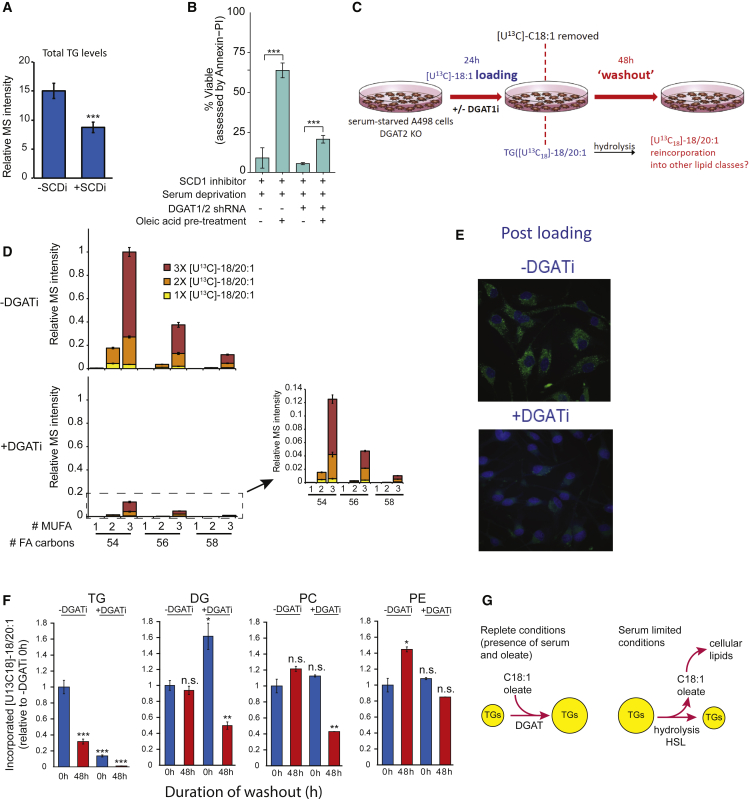
^13^C-Oleate Tracing Reveals a Critical Buffering Role for TG-Resident Unsaturated FAs (A) Effect of SCDi on total TG abundances as measured by LC-MS. (B) Effect of oleate pre-loading with or without *DGAT* shRNA on subsequent A498 cell survival (by Annexin-PI) during serum limitation and SCD inhibition. (C) Schematic of the experimental workflow. *DGAT2* knockout cells were serum-starved for 24 hr and then loaded for 24 hr with 10 μM [U^13^C]-oleate (C18:1) ± DGAT1 inhibitor (T863, 2 μM). The medium was then replaced and the tracer removed, and cells were subjected to a 48-hr washout. (D) TG labeling patterns after 24-hr loading with [U^13^C]-oleate with or without DGATi, where numbers of mono-unsaturated FA (MUFA) and FA carbons are indicated. 1×, 2×, and 3× indicate whether TGs have one, two, or three oleates (includes [^13^C_18_]-20:1) conjugated to their glycerol backbones. (E) BODIPY and DAPI staining directly after [U^13^C]-oleate loading with or without DGATi. (F) Labeling patterns as assessed by incorporation of the ^13^C label in 18:1 and 20:1 FAs in TG, DG, PC, and PE species. (G) Model of the metabolic mechanism by which TGs alleviate the saturation of certain lipid classes (e.g., PCs) under conditions of unsaturated lipid deprivation by releasing stored oleate. Data are means of triplicate wells confirmed in independent experiments (A, B, and D) or means of three independent experiments each conducted in triplicate (F); error bars represent SD. Statistical significance by t test or ANOVA, as appropriate. ^∗^p < 0.05, ^∗∗^p < 0.05, and ^∗∗∗^p < 0.005. See also Figure S3.

Although the aforementioned protective role of TGs could simply be due to their ability to sequester excess saturated FAs into TGs, TG synthesis by DGAT enzymes is more efficient when the substrates are unsaturated rather than saturated (Listenberger et al., 2003). LDs could alternatively protect cells by preferentially releasing unsaturated FAs from stored TGs for use in the production of cytosolic and membrane-associated lipids. Because monounsaturated oleate (C18:1) is the single most abundant FA in TG pools (Figure S3A), its mobilization during periods of unsaturated lipid deprivation should ameliorate stress by preventing the synthesis of fully saturated, potentially toxic lipids. A498 cells experiencing stringent conditions of low serum and pharmacological SCD inhibition exhibited reduced total TG levels (Figure 3A), supporting this hypothesis. To further test this, we assessed the protective potential of oleate, which is efficiently incorporated into TGs and, consequently, the most abundant TG FA. A498 cells were pre-treated with oleate under low-serum conditions before being exposed to SCD inhibition. Oleate pre-treatment indeed promoted cell viability under these conditions (Figure 3B), which was largely abolished upon *DGAT* silencing, suggesting that it occurs through TGs.

To study the cellular fate of oleate in more detail, we designed a washout labeling approach (Figure 3C; see STAR Methods for information about lipid tracing). Serum-starved and, hence, LD-depleted A498 cells were first exposed to labeled [U^13^C]-oleate in the “loading” phase. We employed cells with *DGAT2* loss induced through CRISPR/Cas9-mediated mutagenesis (Figure S1A) to again precisely time DGAT inhibition by adding DGAT1i. Following loading, the [U^13^C]-oleate tracer was removed, and [U^13^C]-oleate in the TG pool was left to “wash out,” allowing its fate (such as re-incorporation into other lipid classes) to be determined. Mass spectrometry analysis of TGs post-loading but pre-washout demonstrated that oleate was avidly incorporated into TGs, mostly producing TGs with three oleate FAs (Figure 3D; i.e., 3× [U^13^C]-18:1 versus 2× or 1× [U^13^C]-18:1). Similarly, BODIPY imaging of neutral lipid stores was used to confirm that oleate loading led to abundant TG accumulation, largely prevented by DGAT inhibition (Figure 3E). Moreover, a non-negligible amount of [U^13^C]-oleate (C18:1) was elongated to [^13^C_18_]-20:1 (i.e., a C20:1 FA with 18 ^13^C) prior to its incorporation into TGs like TG (56:3) (Figure 3D; data not shown). As expected, DGAT inhibition severely limited the incorporation of labeled oleate into TGs.

Based on our data, we reasoned that, under conditions of saturated FA excess, TG oleate mobilization and subsequent re-esterification into other lipid classes enables continued production of lipids with at least one unsaturated FA, preventing the synthesis of harmful, fully saturated lipids. To evaluate the dynamics of oleate redistribution to other lipid species, changes in bulk [U^13^C_18_]-18:1 and [U^13^C_18_]-20:1 in TGs, DGs, and membrane phospholipids, phosphatidylcholines (PCs) and phosphatidyl ethanolamines (PEs), were analyzed after a 48-hr washout in low serum (Figure 3F). In accordance with total TG levels (Figure 3A) and BODIPY imaging (Figure 3E), cells loaded with labeled oleate (0 hr, −DGAT1i) exhibited a strong reduction in TG FA labeling over the course of the washout (48 hr). Cells treated with DGAT1i during loading had lower labeled FA levels to start, which were almost entirely depleted during washout (Figure 3F). Analysis of DG, PC, and PE lipids indicated that [U^13^C_18_] mono-unsaturated FAs (MUFAs) were incorporated into all three classes during loading. Because the exogenous [U^13^C]-oleate tracer was removed at the start of washout, we expected levels of labeled FAs in these lipids (as for TGs) to diminish because of continuous turnover. This was evidently the case for the DGAT1i-treated cells because at least a 2-fold reduction in labeling was observed. Strikingly, this reduction was not detected in untreated cells, most likely because oleate originally loaded in the TG pool is subsequently feeding into DG, PC, and PE lipid pools during washout. By reducing the TG pool, DGAT inhibition prevents the subsequent flow of oleate from TGs to other lipid classes during periods of unsaturated lipid deprivation

Adipose triglyceride lipase (ATGL) and monoacylglycerol lipase (MAGL) have been shown previously to play supportive roles in cancer progression (Nomura et al., 2010, Zagani et al., 2015), suggesting that mobilization of TG stores is critical for tumor cell metabolism. We investigated the outcome of pharmacological inhibition of TG hydrolysis (Figure S3B) and did not observe appreciable effects of disrupting ATGL or MAGL activity on the flow of labeled oleate from TG into other lipid types (see STAR Methods for experimental details). In contrast, inhibition of hormone-sensitive lipase (HSL) led to accumulation of labeled DG, in accordance with its ascribed function as a DG lipase (Figure S3B). HSL inhibition also substantially reduced the washout of TG labeling and caused more abundant PC labeling than in untreated cells. The effects of HSL inhibition were confirmed in two additional ccRCC cell lines (Figure S3C), consistent with DG’s role as a substrate for PL synthesis. Of note, in 786-O cells, almost no labeled FAs were left in the TG pool at the end of the washout experiment, suggesting that the FA buffering capacity in this cell line is somewhat more limited. This is consistent with the more pronounced saturation of the DG pool upon cellular stress (Figure S2F) and our observation that these cells have lower LD numbers under our conditions (data not shown). Our findings demonstrate that oleate (and, to a lesser degree, its elongated C20:1 product) can be released from the TG pool to feed into other lipid classes and most likely requires HSL to break down DGs produced by TG hydrolysis.

Based on our extensive labeling studies of TG turnover under different growth conditions, we propose that TGs act as a buffer for unsaturated FA (mostly oleate; Figure 3D) availability (Figure 3G). During exposure to high serum lipid and/or oleate levels, cells store large amounts of oleate in TGs via DGAT activity. When transitioning to a low-serum environment, TG pools shrink because of HSL-mediated hydrolysis, and released oleate replenishes other lipid species. This helps maintain a viable FA composition when cells are additionally experiencing excess FA saturation, as occurs during tumor hypoxia.

### Compromised FA Buffering by TGs Causes Diversion of Saturated FAs into Toxic Ceramides and Acyl-carnitines

**Figure 4:**
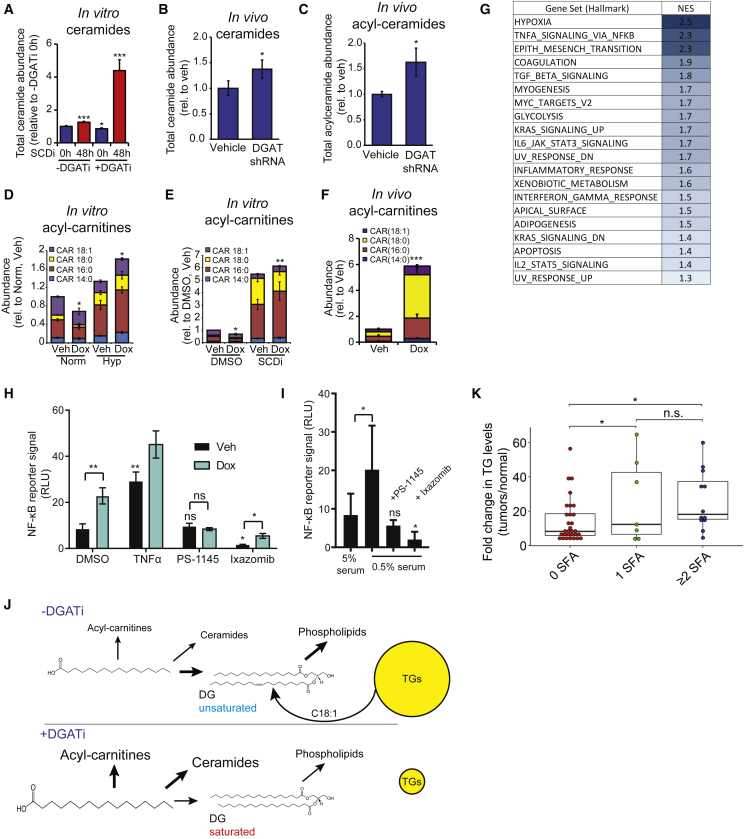
DGAT Loss Modifies Lipid Homeostasis, Elevates Ceramide, Acyl-ceramide, and Acyl-carnitine Levels, and Activates NF-kB Target Gene Expression (A) Effect of SCD and DGAT inhibition on ceramide levels in serum-deprived A498 cells *in vitro*. (B) Effect of DGAT loss on ceramides *in vivo* (i.e., A498 xenografts). (C) Effect of DGAT loss on acyl-ceramides *in vivo* (i.e., A498 xenografts). (D) Effect of hypoxia on the FA composition of acyl-carnitines (CARs) on serum-deprived A498 cells *in vitro*. (E) Effect of DGAT loss on the FA composition of acyl-carnitines (CARs) on serum-deprived A498 cells *in vitro*. (F) Effect of DGAT loss on the FA composition of acyl-CARs in A498 xenograft tumors. (G) Gene set enrichment analysis (GSEA) on RNA as assessed by microarray comparisons performed on A498 *DGAT* shRNA xenograft tumors after 5 days of control or doxycycline chow. Normalized enrichment score (NES) allow comparison of enrichment between different gene sets. (H) Effect of *DGAT* shRNA, NF-κB inhibition, and proteasome inhibition on NF-κB luciferase reporter activity. (I) Effect of serum deprivation on NF-κB luciferase reporter activity. (J) Schematic of the consequences of DGAT inhibition preceding a period of unsaturated lipid deprivation. These conditions lead to increased acyl-CARs and ceramides as well as increased incorporation of saturated FAs into the PL pool. (K) Published data comparing the TG composition of ccRCC and normal tissue (Saito et al., 2016) were reanalyzed to investigate shifts in TG saturation. TG species were binned according to the number of fully saturated FA chains present, and the abundance of each category was aggregated and is displayed as a ratio of the abundance in normal tissue. For (A), (D), (E), data are means of 5 and for (H) and (I) of 3 replicate wells, and results were confirmed in independent experiments. For (B), (C), (F), and (G), data are means of tumors from 4 tumors. Error bars represent SD. Statistical significance by t test or ANOVA, as appropriate. ^∗^p < 0.05, ^∗∗^p < 0.05, and ^∗∗∗^p < 0.005. See also Figure S4.

Because ceramides are predominantly generated from saturated FAs, and excess buildup promotes apoptosis, we investigated their abundance following DGAT inhibition. DGAT depletion resulted in a large increase in ceramide levels in A498 cells treated with SCD inhibitor under serum-deprived conditions (Figure 4A) and A498 xenograft tumors (Figure 4B), along with acyl-ceramides (Figure 4C). Acyl-carnitines have recently been shown to be elevated upon DGAT inhibition, contributing to mitochondrial dysfunction (Nguyen et al., 2017). In line with this, we observed elevated acyl-carnitine upon DGAT knockdown in the context of *in vitro* hypoxia (Figure 4D), *in vitro* SCD inhibition (Figure 4E), and *in vivo* xenograft tumors (Figure 4F). These changes indicate that disrupted TG synthesis widely affects lipid homeostasis and accumulation of toxic lipid species.

To further evaluate the effects of decreased cellular lipid homeostasis through DGAT loss, we performed both *in vivo* and *in vitro* microarray studies. A498 tumor samples harboring *DGAT* shRNA were compared with controls, and a gene set enrichment analysis (GSEA) was performed (Figure 4G). A parallel analysis of A498 cells grown under 0.5% serum and exposed to either 21% O_2_ or 0.5% O_2_ for 48 hr, with or without induction of *DGAT* shRNA expression, was also performed (Figures S4A and S4B). The expression of transcriptional targets of nuclear factor κB (NF-κB) exhibited a striking pattern of regulation that suggests induction by lipid dysregulation. *In vivo*, DGAT depletion led to a significant increase in NF-κB target gene expression (Figure 4G). *In vitro*, the same gene set was enriched by hypoxia, DGAT loss, and the combination of these two treatments under serum deprivation (Figures S4A–S4C). Of note, we observed a stepwise increase in many NF-κB target gene mRNAs by sequentially combining DGAT loss and hypoxia (Figure S4D). NF-κB is known to be engaged under conditions of stress and excess saturated FA exposure (Van Beek et al., 2012, Carluccio et al., 1999, Massaro et al., 2002), regulating desaturase activity and ovarian cancer stem cell identity (Li et al., 2017). We therefore hypothesized that the elevated lipid saturation detected in DGAT-depleted tumors engages the NF-κB pathway. Using a luciferase reporter, we confirmed that both basal and tumor necrosis factor α (TNF-α)-induced NF-κB activity was enhanced by DGAT loss in A498 cells (Figure 4H). Importantly, NF-κB pathway engagement was inhibited by the specific NF-kB inhibitor PS-1145 (Figure 4H) as well as the proteasome inhibitor ixazomib. Of note, ixazomib inhibits NF-κB by stabilizing its negative regulator inhibitor of κB (IκB) (Mujtaba and Dou, 2011). Serum deprivation also led to increased NF-κB reporter activity, which was fully inhibited by administration of both PS-1145 and ixazomib (Figure 4I). Collectively, our data suggest that DGAT deficiency leads to broad disruption of lipid homeostasis, resulting in unsaturated FA depletion and accumulation of saturated FAs and, consequently, ceramides, acyl-ceramides, and acyl-carnitines (Figure 4J). This is accompanied by activation of NF-κB signaling, previously documented to respond to increased saturated FAs. Future work will delineate whether NF-κB signaling contributes to balancing FA availability during stressful conditions as a prosurvival mechanism.

### Altered TG Saturation in ccRCC Patient Samples

Given the requirement for DGAT activity to survive conditions of excess lipid saturation in our experiments, we determined whether this is a common feature of human tumor biology. We therefore re-analyzed published data comparing the TG composition of ccRCC patient samples and normal kidney tissues (Saito et al., 2016). As expected, TG levels were strongly elevated in ccRCC, as documented previously (Sundelin et al., 2012). However, the fold change was particularly pronounced for TGs containing one or more SFAs, demonstrating an increase in TG saturation (Figure 4K). This increase in ccRCC TG saturation is consistent with TG regulation of FA composition in lipid pools, as observed in our *in vitro* experimental system (Figures 3E and 3F). We therefore suggest that increased FA saturation (and its buffering by TGs) occurs in tumor samples derived from ccRCC patients.

### Conclusion

Although oncogenic signaling alters cellular metabolism to promote the synthesis of macromolecules, malignant cells are also programmed to withstand nutrient scarcity (Boroughs and DeBerardinis, 2015). This metabolic flexibility can involve the induction of scavenging pathways, such as autophagy in RAS- or BRAF-driven cells (Yang et al., 2011) or macropinocytosis in KRAS-driven cells deprived of glutamine (Commisso et al., 2013). Both KRAS and hypoxia promote the uptake of extracellular unsaturated lipids with similar consequences: cells become more resistant to O_2_ deprivation and its associated inhibition of FA desaturases (Kamphorst et al., 2013). Here, in the case of LDs, hydrolysis of unsaturated TGs supplies unsaturated DGs and FAs as substrates for PL synthesis, maintaining lipid homeostasis during periods of increased FA saturation. We therefore propose that unsaturated FA storage in TGs contributes to ccRCC metabolic plasticity and that its inhibition may effectively target cancer cells residing in ischemic tumor domains (Figure 4J).

## STAR★Methods

### Key Resource Table

**Table:** 

REAGENT or RESOURCE	SOURCE	IDENTIFIER
**Antibodies**		

DGAT1	Abcam	ab54037; RRID: AB_869453
V5	Life Technologies	R960-25; RRID: AB_2556564
KI67	Abcam	ab15580; RRID: AB_443209
Cleaved Caspase3	Cell Signaling	9661; RRID: 2341188
Calnexin	Cell Signaling	2679; RRID: 10827903)

**Chemicals, Peptides, and Recombinant Proteins**		

DMEM	Life Technologies	11965-084
Pen/Strep	Life Technologies	15140-122
Standard FBS	Gemini	900-108
[U^13^C]-oleate	Sigma	490431
T863 DGAT1i	Sigma	SML0539-5MG
Matrigel Basement Membrane Matrix	Corning	356234
200mg/kg doxycycline chow	Harlan Labs	TD04104
oleic acid- BSA mix	Sigma	O3008
Butylated hydroxytoluene (BHT)	Sigma	W218405
SPLASH lipidomix internal standard mix	Avanti Polar Lipids	330707
Atglistatin	Sigma	SML1075
CAY10499	Cayman chemicals	10007875
JJKK048	Tocris	5206

**Critical Commercial Assays**		

Volupac	Sartorius	11729265
RNAeasy purification kit	QIAGEN	74106
High Capacity RNA-to-cDNA master mix	Life Technologies	4387406
TBP Taqman assay	Life Technologies	HS00427620_M1
ACTB Taqman assay	Life Technologies	HS01060665_G1
DGAT1 Taqman assay	Life Technologies	HS01017541_M1
DGAT2 Taqman assay	Life Technologies	HS01045913_M1
QiaPrep Miniprep kit	QIAGEN	27104
BODIPY 493/503	Life Technologies	D3922
FITC–Annexin V, PI Kit	BD Biosciences	556547
Annexin-V binding buffer	BD Biosciences	556454

**Deposited Data**		

*In vivo* microarray study	NCBI GEO	GSE117774
*In vitro* microarray study	NCBI GEO	GSE117775

**Experimental Models: Cell Lines**		

A498	ATCC	HTB-44
786-O	ATCC	CRL-1932

**Experimental Models: Organisms/Strains**		

NIH-III nude mice (female) 4-6 weeks old	Charles River	#201

**Oligonucleotides**		

DGAT2 Crispr1:	This paper	N/A
Forward: caccgTGTGCTCTACTTCACTTGGC		
Reverse: aaacGCCAAGTGAAGTAGAGCACA		
DGAT2 Crispr2:	This paper	N/A
Forward: caccgGTACATGAGGATGGCACTGC		
Reverse: aaacGCAGTGCCATCCTCATGTAC		
TCCTCTTGTCCCAGGAATCTGC	This paper	DA182
CACTCAGGATGAGGCCCTTCAG	This paper	DA184
GAATCTGCTCCTACCTGGGCTG	This paper	DA183
GTTTCTgctagcATGAAGACCCTCATAGCCGC	This paper	DA199
GTTTCTgcggccgcTCAATGGTGATGGTGATGATG	This paper	DA200
gtttctGCGGCCGCtcaGTTCACCTCCAGGACCTCAG	This paper	DA201
TACTGGGAGTGGCaTGCAGTGCCAT)	This paper	DA187
ATGGCACTGCAtGCCACTCCCAGTA).	This paper	DA188

**Recombinant DNA**		

pLKO.1 Scramble	Addgene	17920
shDGAT2_5	GE Dharmacon	TRCN0000005195
shDGAT1_1	GE Dharmacon	TRCN0000036151
Tet-pLKO-puro	Addgene	21915
Tet-pLKOneo	Addgene	21916
lentiCrisprv2	Addgene	98290
DGAT2 cDNA in pcDNA3.1 vector	GeneCopoeia	T7986
pCDH-CMV-MCS-EF1-Neo	System Biosciences	CD514B-1

**Software and Algorithms**		

LipidSearch	Thermo Fisher Scientific/ Mitsui Knowledge Industries	IQLAAEGABSFAPCMBFK
MAVEN		http://genomics-pubs.princeton.edu/mzroll/index.php
GraphPad Prism 7.0	GraphPad Software	https://www.graphpad.com/scientific-software/prism/

### Contact for Reagent and Resource Sharing

Further information and requests for resources and reagents should be directed to and will be fulfilled by the Lead Contact, Jurre Kamphorst (jurre.kamphorst@glasgow.ac.uk).

### Experimental Model and Subject Details

#### Mice

Subcutaneous xenograft experiments were approved by the Animal Care and Use Committee at the University of Pennsylvania. 10 Female NIH-III nude mice (Charles River; 4–6 weeks old) were injected subcutaneously in both flanks. The injection mix contained 5 million cells in PBS, mixed 1:1 with Matrigel Basement Membrane Matrix (Corning). Tumor volume was monitored by caliper measurements. After tumors reached 300 mm3, mice were split into cohorts of 5 mice receiving doxycycline chow (200 mg/kg; Harlan Labs) or control chow (Harlan Labs) *ad libitum*. After experiment completion, animals were sacrificed by CO2 inhalation, and xenograft tumors were dissected for downstream analyses.

#### Cell Lines and cell culture conditions

Authenticated (short tandem repeat profiling) human cell lines HK-2, 786-O, 769-P, A498, RCC4, RCC10, UOK101, and UMRC2 were obtained from the American Type Culture Collection. Cell lines were routinely passaged in DMEM (GIBCO) with 25 mM glucose and 2 mM L-glutamine with 5% (v/v) fetal bovine serum (FBS, GIBCO) at 37°C and 5% CO_2_. Cells were split at 80% confluency. All cell lines described in this study were verified mycoplasma-negative. Experiments were performed in DMEM supplemented with 10 mM glucose, 2 mM L-glutamine and indicated levels of FBS (Sigma). Hypoxic conditions were maintained at 0.5% O_2_, 37°C and 5% CO_2_ in the InVivO_2_ Hypoxia Workstation with a Ruskinn Gas mixer Q (Baker Co.). Cell number was assessed using the Countess Cell Counter (ThermoFisher) or estimated using packed cell volume (PCV, Sartorius Volupac), as appropriate.

### Method Details

#### ^13^C-FA tracing

For the [U^13^C]-oleate washout experiment, DGAT2 knockout A498 cells were seeded in 6-well plates and serum starved in DMEM containing 0.5% dialyzed FBS for 24h. The medium was then replaced with new medium containing 10 μM [U^13^C]-oleate (Sigma) with or without DGAT1i (T863, 1 μM) and incubated for 24h. The medium containing [U^13^C]-oleate was replaced with fresh medium (0.5% dFBS). Cells were incubated for 48h in these conditions followed by lipid extraction as described in Lipid extraction and liquid chromatography - mass spectrometry (LC-MS) analysis, below. The tracing experiment was repeated three times independently with each condition conducted in triplicate.

#### qRT-PCR studies

Total RNA was isolated using the RNAeasy purification kit (QIAGEN). cDNA was synthesized using the Applied Biosystems High Capacity RNA-to-cDNA master mix. qRT-PCR was performed on a ViiA7 Real Time PCR systems from Applied Biosystems. Pre-designed Taqman primers were obtained from Life Technologies for the following genes: TBP (HS01060665_G1), ACTB (HS01060665_G1), DGAT1 (HS01017541_M1), and DGAT2 (HS01045913_M1).

#### DGAT mutant and knockdown lines

*DGAT1* and *DGAT2* shRNA (specified as *DGAT* shRNA unless stated otherwise) was achieved by expressing Dox-inducible sh*DGAT2_5* (TRCN0000005195) using the Tet-pLKO-puro plasmid and sh*DGAT1_1* (TRCN0000036151) using the Tet-pLKO-neo. Lentivirus was generated for each plasmid in HEK293T cells and used to infect the relevant cell line. After selection with puromycin and G418, the knockdown of both *DGAT1* and *DGAT2* transcripts was confirmed by qRT-PCR (Taqman probes; ThermoFisher) *DGAT2* knockout cell lines were generated by cloning sgRNA sequences 5′-TGTGCTCTACTTCACTTGGC-3′ and 5′-GTACATGAGGATGGCACTGC-3′ into the lentiviral vector lentiCrisprv2 (Addgene), generating lentivirus in HEK293T cells and transducing ccRCC cell lines with 25μl of un-concentrated supernatant. After puromycin selection, single cell clones were generated by limiting dilutions in 96 well plates. Single-cell clones were expanded and genomic DNA was isolated from a portion of the expanded cell population. PCR of the DGAT2 locus was performed using DA182 (TCCTCTTGTCCCAGGAATCTGC) forward and DA184 CACTCAGGATGAGGCCCTTCAG reverse primers. PCR products were TOPO-cloned using the Zero Blunt PCR cloning kit (ThermoFisher) and transformed into competent *E. coli*. For each clone, 3-6 colonies were picked and grown overnight in LB-Ampicillin. Plasmid DNA was isolated using the QiaPrep Miniprep kit (QIAGEN) and sequenced using a nested primer DA183 (GAATCTGCTCCTACCTGGGCTG). Clones containing mutations in both alleles were tested phenotypically by Bodipy neutral lipid staining, and LC/MS confirmed reduced TG production (data not shown), as expected. Cells were incubated with 1:50 dilution of oleic acid-

BSA mix (Sigma; 2 mole OA/mole albumin; 100mg/ml albumin) for 16h with and without the presence of 2 μM DGAT1i (T863, Sigma). Clonal lines with full loss of DGAT2 activity loss had complete abrogation of neutral lipid storage by oleic acid stimulation when DGAT1 was inhibited. Rescue of DGAT2 loss was performed by cloning DGAT2 cDNA from the pcDNA3.1 vector (GeneCopoeia) into the pCDH lentiviral expression plasmid *pCDH*-CMV-MCS-EF1-*Neo* using the primers DA199 *(*GTTTCTgctagcATGAAGACCCTCATAGCCGC), DA200 (GTTTCTgcggccgcTCAATGGTGATGGTGATGATG) as well as DA199 and DA201 (gtttctGCGGCCGCtcaGTTCACCTCCAGGACCTCAG) for expression with and without V5 and Histags. gRNA sites were then mutated to prevent cutting by Cas9 protein expressed in the mutant cell lines. Synonymous mutations were introduced using DA187 (TACTGGGAGTGGCaTGCAGTGCCAT) and DA188 (ATGGCACTGCAtGCCACTCCCAGTA).

#### Flow cytometry assays

For experiments, cells were seeded in 6-well plates 24 hours before the experiment at a cell density that led to 80% confluency at the end of the experiment. BODIPY 493/503 (Cat D3922) was purchased from Life Technologies and FITC–Annexin V, PI Kit (cat. 556547) from BD Biosciences. Live cells were washed twice in PBS and incubated in 2 μg/ml BODIPY in PBS for 15 minutes at 37°C. After staining, cells were washed twice in PBS and re-suspended in Annexin-V binding buffer (BD Cat 556454), passed through a cell strainer, and analyzed on an Accuri C6 flow cytometer. For viability assays, cells were stained with FITC-Annexin V and PI according to the manufacturer’s instructions and double-negative cells were deemed viable. Median signal intensity for each well was average for triplicate samples to determine staining intensity.

#### Lipid droplet imaging

Cells were seeded on round glass coverslips of 24-well plates and supplemented with 1 mL of medium and exposed to the indicated conditions. The medium was then aspirated, cells washed once with 1 mL room temperature PBS, fixed with 0.5 mL of 4% formaldehyde (Sigma) for 30 min after which excess was removed and cells washed 3x with 1 mL PBS. Cells then were incubated with 0.3mL of 1 μM BODIPY 493/503 (Life Technologies) (excitation wavelength 480nm, emission maximum 515 nm) for 15 min in the dark, washed 2x with 1 mL PBS, incubated with 0.3 mL of 1 μg/mL DAPI (Sigma) for 15 min in the dark and washed 2x with 1 mL PBS. Thereafter, the coverslips were mounted on glass slides using Dako Fluorescent Mounting Medium (Dako). Z stack images were acquired using Olympus FV1000 confocal laser scanning microscope (405 nm laser for DAPI and 488 nm laser for BODIPY) and processed with ImageJ software.

#### Lipid extraction and liquid chromatography - mass spectrometry (LC-MS) analysis

For cultured cells the medium was aspirated and cells washed 2x with 1 mL room temperature PBS. The cells were placed on ice and quenched with 0.75 mL of methanol/PBS (1:1, v/v) at −20°C, and kept for 10 min. The cells were then scraped into glass tubes (Fisher Scientific), 0.5 mL chloroform at −20°C (Sigma) and 50 μL of 1 mg/mL methanolic butylated hydroxytoluene (BHT, Sigma) added, followed by addition of SPLASH lipidomix internal standard mix (Avanti Polar Lipids) at 1 μL per 1^∗^10^5^ cells. This was vortexed for 1 min and centrifuged at 500*g* for 10 min. The chloroform layer was transferred to a new glass vial, dried under nitrogen gas and stored at −20°C for further LC-MS analysis. Samples were reconstituted in chloroform/methanol (1:1 v/v) at 50 μL per 1^∗^10^5^ cells prior to the LC-MS analysis.

For extraction of tumor tissues, 10-35 mg was transferred to ice-cold Precellys lysing tubes, 0.75 mL of methanol/PBS (1:1, v/v) at −20°C and 50 μL of 1 mg/mL BHT in methanol added, and homogenized using pre-cooled Precellys Tissue Homogenizer at −10°C. The homogenization program included 3 cycles of 30 s of shacking at 5,000 rpm and 15 s pause per cycle. Further sample treatment was as for cultured cells, with addition of 0.5 mL chloroform at −20°C and internal standard mix at 10 μL per 10 mg tissue. Samples were reconstituted at a concentration of 200 μL per 10 mg of tissue in chloroform/methanol (1:1 v/v) prior to the LC-MS analysis.

Lipidomic analysis was performed using a Q Exactive orbitrap mass spectrometer coupled to a Dionex UltiMate 3000 LC system (Thermo Scientific). The LC parameters were as follows: 4 μL of sample was injected onto a 1.7 μm particle 100 × 2.1mm ID Waters Acquity CSH C18 column (Waters) which was kept at 50°C. A gradient of (A) water/acetonitrile (40:60, v/v) with 10 mM ammonium formate and (B) acetonitrile/2-propanol (10:90, v/v) with 10 mM ammonium formate at a flow rate of 0.3 mL/min was used. The gradient ran from 0% to 40% B over 6 min, then from 40% to 100% B in the next 24 min, followed by 100% B for 4 min, and then returned to 0% B in 2 min where it was kept for 4 min (40 min total). Lipids were analyzed in both positive and negative mode. The electrospray and mass spec settings were as follows: spray voltage 3 kV (positive mode) and 3.5 kV (negative mode), capillary temperature 300°C, sheath gas flow 50 (arbitrary units), auxiliary gas flow 7 (arbitrary units) and sweep gas flow 5 (arbitrary units). The mass spec analysis was performed in a full MS and data dependent MS^2^ (Top 10) mode, with a full scan range of 300-1200 m/z, resolution 70,000, automatic gain control at 1x10^6^ and a maximum injection time of 250 ms. MS^2^ parameters were: resolution 17,500, automatic gain control was set at 1x10^5^ with a maximum injection time of 120ms.

#### Lipase inhibitor assays

A498, 786-O or UMRC2 cells were grown in 0.5% serum (low serum) for 24h, then loaded with 10 μM U-13C oleate for 24h incubation under low serum. Labeled oleate was washed out by growing cells under low serum for 48h with or without addition of a lipase inhibitor. ATGL inhibitor atglistatin (Sigma SML1075) was used at 50 μM, HSL inhibitor CAY10499 (Cayman chemicals) was used at 50 μM and MAGL inhibitor JJKK048 (Tocris) was used at 50 μM. Cells were then counted and harvested for lipidomic analysis.

#### Microarray experiments

For *in vivo* analysis of gene expression following inducible DGAT knockdown, mice bearing 300mm3 tumors from subcutaneously injected A498 *DGAT* shRNA cells were fed either doxycycline- or control- chow for 5 days. The animals were then sacrificed, the tumors harvested and RNA was extracted using the RNEasy kit (QIAGEN). RNA was then deposited with the University of Pennsylvania Molecular Profiling core facility for processing, microarray analysis using the Affymetrix HTA 2.0 Chip and analysis.

#### Suitability of using ^13^C-labeled FAs to study lipid metabolism

FAs supplied to cells can be used for oxidation to generate energy, can directly be incorporated into lipids, or can first be further matured (i.e., elongated, desaturated) prior to lipid assembly. Oxidation of ^13^C-labeled FAs leads to generation of ^13^C-acetyl-CoA, which in turn can be used for the synthesis of new FAs. This would lead to complex labeling distributions that would complicate interpretation of labeling patterns and hence lipid metabolic events. To determine the feasibility of using ^13^C-FAs to study lipid metabolism, A498 cells were incubated for 6 hours with 25 μM [U^13^C]-stearate (C18:0) and labeling of triglycerides was assessed (Figure S5A). This short time span was sufficient to generate extensively labeled TGs. Notably, after correcting for natural ^13^C occurrence, the majority of TG isotopologs observed were the unlabeled (M^0^), the M^+18^ isotope corresponding to the incorporation of one [U^13^C]-C18:0, as well as M^+36^ and M^+54^ that result from the incorporation of 2 and 3 labeled FAs, respectively. Some minor odd-labeled isotopes (M^+19^, M^+37^, M^+55^) were observed; these are most likely caused by imperfect corrections for ^13^C-natural abundance by the algorithm. Importantly, no significant partial labeling was observed, demonstrating that FA synthesis from ^13^C-acetyl-CoA due to FA oxidation is not detectable and does not complicate FA tracing experiments.

Fragmentation spectra of labeled TGs further confirmed that labeled FAs shorter than 18 carbons do not occur. This is arguably best demonstrated by the MS^2^ pattern of TG(48:0) M^+18^ (one labeled FA, Figure S5B). While one could assume that TG(48:0) is primarily made up of 3x C16:0 (palmitate), the fragmentation pattern actually reveals a mixture of FA compositions, which each combination totaling 48 carbons (16:0/16:0/16:0, 16:0/18:0/14:0, 12:0/18:0/18:0). This means that these TGs have the same mass and do not separate by LC-MS. While this should be kept in mind, it does not affect interpretation of the labeling pattern. Importantly, in TG(48:0) M^+18^ and other TGs (data not shown) only [U^13^C]-C18:0 is observed and no shorter ^13^C-FAs, further demonstrating that partial oxidation of labeled FAs does not occur in these cells.

We did find that Labeled stearate is desaturated and elongated leading to FAs such as [U^13^C]-18:1 and [^13^C_18_]-20:0 as well as longer chain FAs, as evidenced by the direct observation of their acylium ions in MS^2^ (FAs are observed as their acylium ions in positive mode MS^2^, Figure S5C). We therefore concluded that tracing with ^13^C-labeled FAs is suitable for investigating the dynamics of lipid metabolism.

### Quantification and Statistical Analysis

#### Lipidomic data processing

Peak detection, peak area quantification, lipid identification, and alignment were performed using LipidSearch (Thermo Fisher Scientific/Mitsui Knowledge Industries) with standard settings for Q Exactive Product Search. Data was then exported to Excel and lipid peak areas were normalized to the peak area of the corresponding lipid internal standard using an in-house R script. The normalized peak areas of identified lipids were used for plotting.

Volcano plots were generated using *ggplot2* R package by plotting *log* Fold change (n = 5 for each condition) against *–log* P value (Wickham, 2009). Significant changes with ≥ 1.5-fold and p ≤ 0.05 are indicated in color according to the figure legend.

Saturation indices for different lipid classes are represented as a ratio of total palmitate and stearate to oleate. The total level of palmitate in individual lipid class was calculated by summing up the intensities of each palmitate-containing lipid multiplied by the number of palmitate moieties in each lipid (i.e., total palmitate in TG = Σ (1^∗^TG(*16:0*/18:0/18:1) + 2^∗^TG(*16:0*/*16:0*/18:1) +3^∗^TG(*16:0*/*16:0*/*16:0*))). The same principle was used for calculation of total stearate and oleate.

For stable isotope tracing experiments MAVEN software was used. A total ^13^C-FA incorporation value for each lipid class was calculated by summing up the labeling intensities for those lipids of that class that were most intensely labeled and together contained ≥ 80% of total label. A labeling intensity per lipid was calculated by summing up the intensities for each labeled isotope multiplied by the number of labeled FAs for that particular isotope (i.e., total labeling = Σ (1^∗^M+18 + 2^∗^M+36 +3^∗^M+54)).

#### Statistical Analysis

For bar plot, the height of the bar represents the mean of all replicates and error bars represent ± SD or ± SEM, as indicated in the Figure Legends and Supplemental Figure Legends. Replicate numbers, statistical tests used and explanations for error bars are indicated in the Figure Legends and Supplemental Figure Legends. Statistical significance was derived using R or GraphPad Prism 7.0 by t test or ANOVA, as appropriate; ^∗^p < 0.05, ^∗∗^p < 0.01 and ^∗∗∗^p < 0.001.

### Data and Software Availability

The A498 DGAT shRNA *in vivo* microarray experiment and the A498 DGAT shRNA *in vitro* microarray experiment data were deposited at NCBI GEO (https://www.ncbi.nlm.nih.gov/geo/) under accession numbers GEO: GSE117774 and GSE117775 respectively.

## Supplemental Information

Document S1. Figures S1–S5

Table S1. Raw Lipidomics Data of *In Vitro* and *In Vivo* Studies, Related to Figure 1

Document S2. Article plus Supplemental Information
