# Supplementary material for: Triglycerides Promote Lipid Homeostasis during Hypoxic Stress by Balancing Fatty Acid Saturation
Source: Cell Rep. 2018 Sep 4;24(10):2596–2605.e5. doi: 10.1016/j.celrep.2018.08.015 (PMC6137821; doi:10.1016/j.celrep.2018.08.015)
Supplement: Document S1. Figures S1–S5 [file mmc1.pdf]

Cell Reports, Volume *24*

Supplemental Information

Triglycerides Promote Lipid Homeostasis during Hypoxic Stress by Balancing Fatty Acid Saturation

Daniel Ackerman, Sergey Tumanov, Bo Qiu, Evdokia Michalopoulou, Michelle Spata, Andrew Azzam, Hong Xie, M. Celeste Simon, and Jurre J. Kamphorst

Supplemental Figures

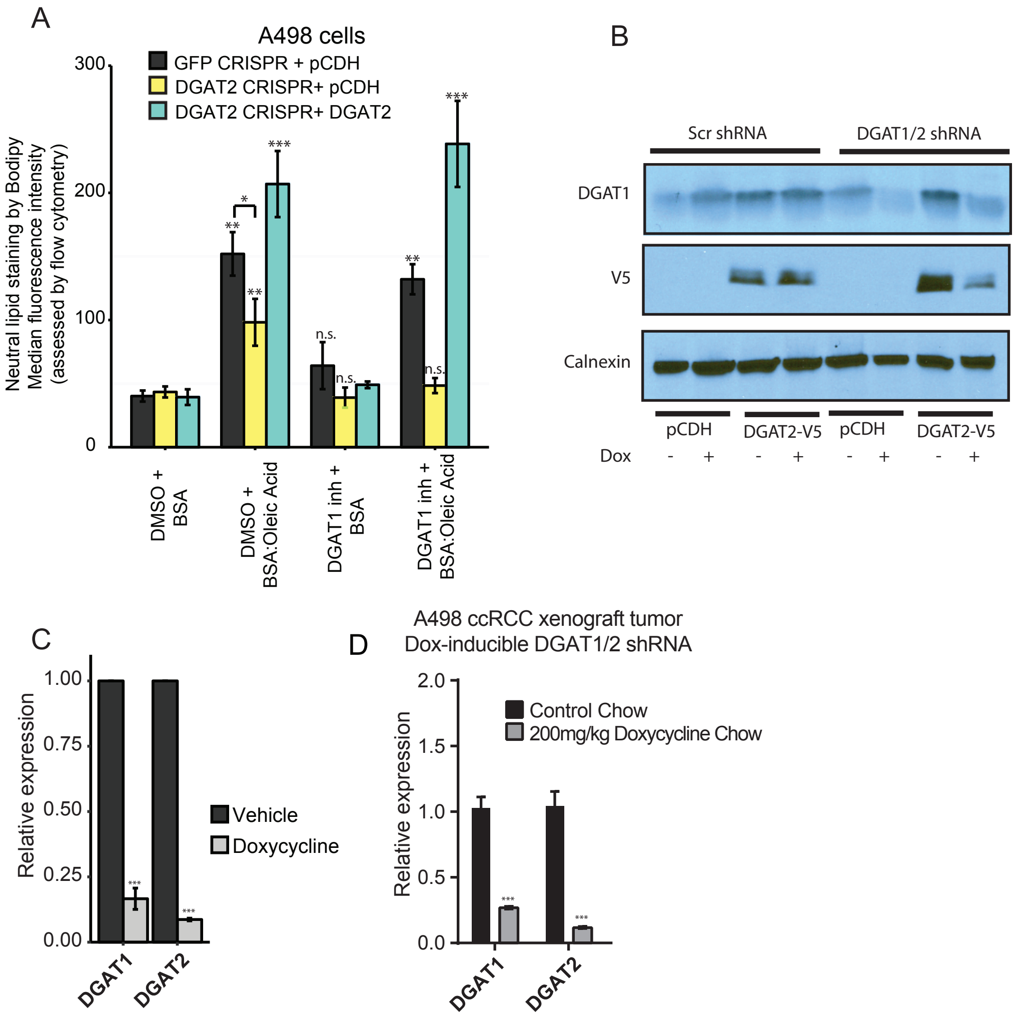

Figure S1. Related to Figure 1.
(A) DGAT2 knockout A498 cells and GFP control cells, with and without DGAT1 inhibitor treatment, were incubated with an excess of oleic acid conjugated to BSA and the induction neutral lipid storage assessed by flow cytometry using BODIPY. BSA and DMSO serve as FA carrier and inhibitor vehicle controls, respectively. Specificity of DGAT1/2 inhibition was confirmed by complementation with pCDH vector expressing CRISPR-resistent DGAT2 cDNA versus pCDH vector controls. (B) Due to unreliable antibodies against DGAT2, efficacy of DGAT1/2 shRNA against DGAT2 mRNA was tested using a DGAT2-V5 transgene and blotted against the V5 tag. Cells were treated with Vehicle or Doxycycline for 5 days before lysis and western blotting analysis. (C) qRT-PCR on RNA extracted from A498 cells expressing dox-inducible DGAT1/2 shRNA after 48h of doxycycline treatment. (D) qRT-PCR on RNA extracted from tumors after 9 days of ad libitum Doxycycline chow at 200mg/kg. For (A), (C) data are means of triplicate wells and were confirmed in independent experiments, and for (D) data are means of 10 tumors, derived from 5 mice harbouring bilateral flank tumors per group; error bars represent SD. Statistical significance by t-test or ANOVA, as appropriate. *: $p<0.05$, **: $p<0.01$ and ***:$p<0.001$.

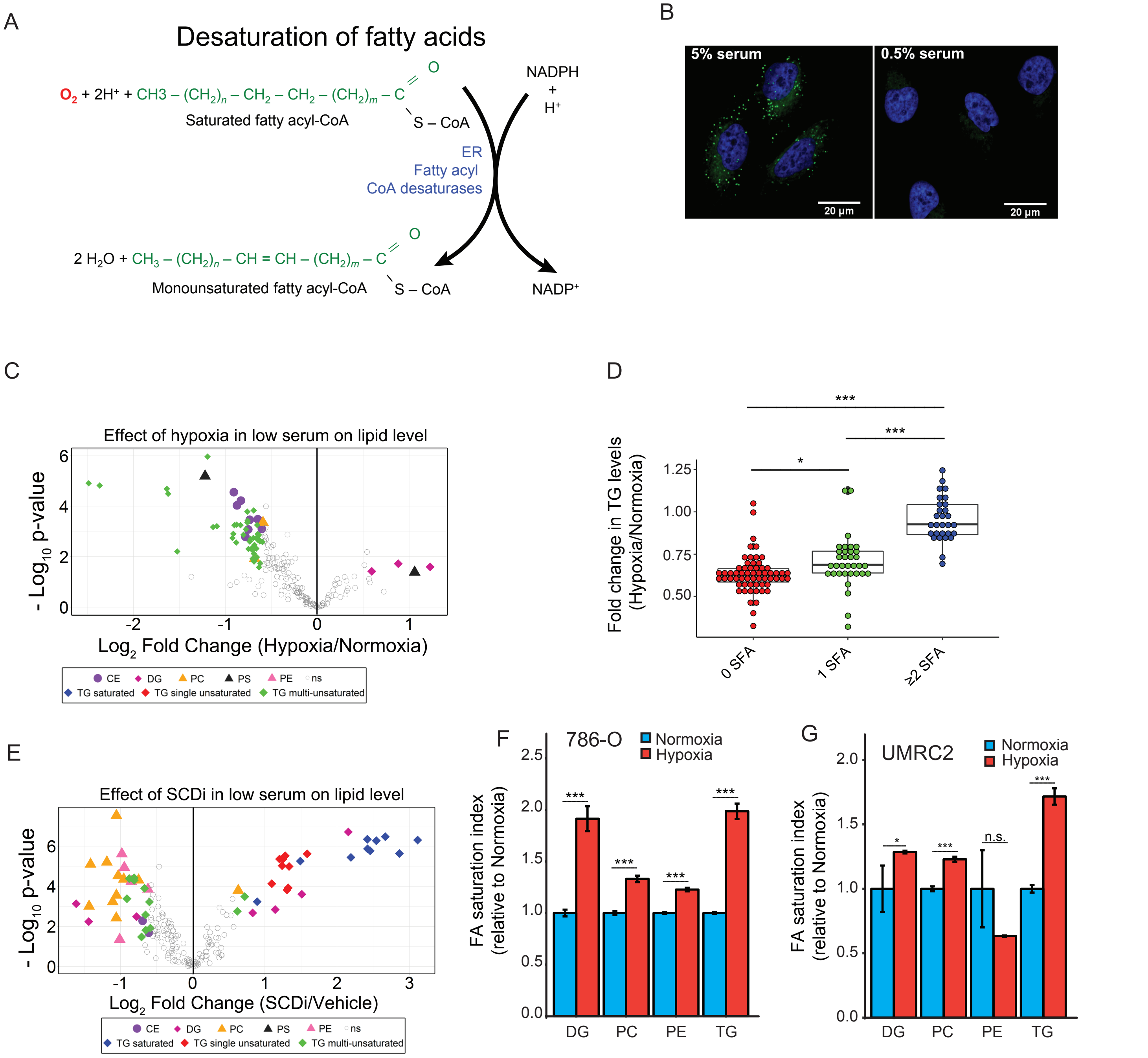

Figure S2. Related to Figure 2.
(A) Diagram of the oxygen (O2)-dependent desaturation of FAs by SCD enzyme in the ER. (B) Confocal microscopy images of cells in 5% and 0.5% serum with neutral lipid staining by BODIPY 493/503 (green) and DNA by DAPI (blue). (C) Volcano plot showing fold-change and significance of alterations in the lipidome of A498 cells cultured in hypoxia (0.5% O2) versus normoxia. Lipids with ≥ 1.5 fold change and p ≤ 0.05 are displayed in a color to denote lipid class. (D) TG species binned according to the number of fully saturated FA (SFA) chains present and the abundance of each category aggregated and displayed as a ratio of the abundance in hypoxia compared to the control group (normoxia). (E) Volcano plot showing fold-change and significance of alterations in the lipidome of A498 cells cultured with 1 μM SCD inhibitor (CAY10566, Cayman Chemicals) vs DMSO control. Lipids with ≥ 1.5 fold change and p ≤ 0.05 are displayed in a color to denote lipid class. (F) Lipid class-specific saturation indices, for 786-O cells cultured at 0.5% O2 relative to untreated vehicle control. (both in low serum). (G) as in (F,) but using UMRC2 ccRCC cells. Data are means of 3 (D, F, G) or 5 (D, E) replicate wells and were confirmed in independent experiments; error bars represent SD. Statistical significance by t-test or ANOVA, as appropriate. *: p<0.05, **: p<0.05 and ***:p<0.005.

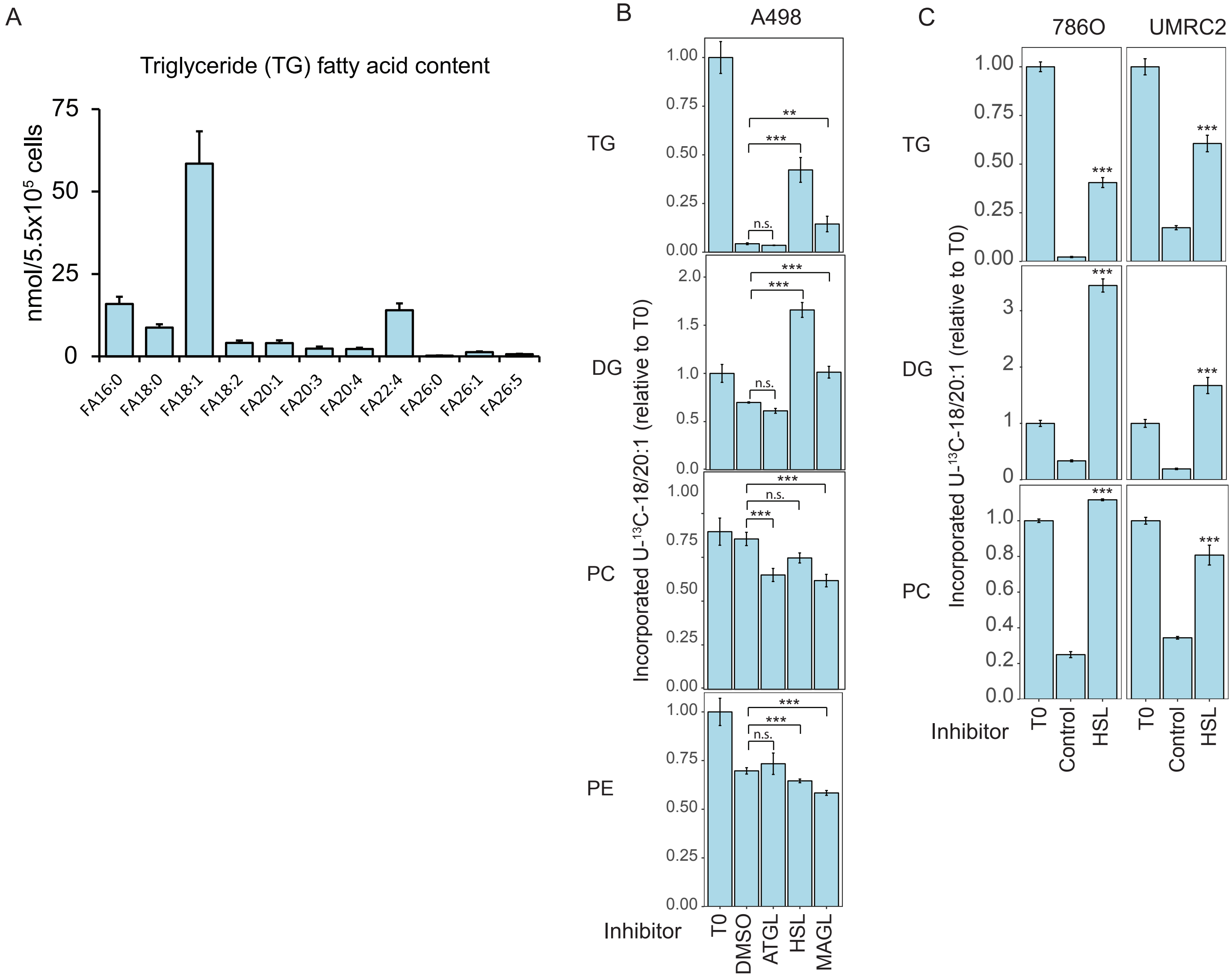

Figure S3. Related to Figure 3.

(A) FA composition of TG pools derived from lipidomic data of untreated A498 cells. (B) A498 cells preloaded with [U13C]-Oleate (C18:1) for 24h, and subsequently treated with low serum and DMSO vehicle or one of several lipase inhibitors for 48h. The proportion of labeled FA of various lipid types was assessed by LC-MS. (C) Like (B), but in 786-O and UMRC2 cells using only the HSL inhibitor. The concentration of ATGL, HSL and MAGL inhibitors used was 50 μM. Data are means of triplicate wells and were confirmed in independent experiments; error bars represent SD. Statistical significance by t-test or ANOVA, as appropriate. *: $p<0.05$, **: $p<0.05$ and ***:$p<0.005$. Abbreviations: ATGL – adipose triglyceride lipase, HSL - hormone sensitive lipase, MAGL – monoacylglycerol lipase. T0- time zero (i.e. start of the experiment).

A

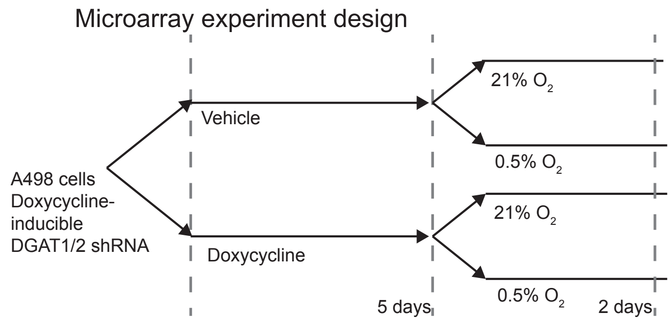

B

Gene Set (Hallmark)	DGAT1/2 shRNA effect	Hypoxia control	Hypoxia DGAT1/2 shRNA
TNFA_SIGNALING_VIA_NFKB	2.5	1.5	1.7
HYPOXIA	2.1	1.4	1.9
MITOTIC_SPINDLE	0.0	2.7	2.6
G2M_CHECKPOINT	0.0	2.2	2.2
E2F_TARGETS	0.0	1.9	1.9
PROTEIN_SECRETION	1.9	2.4	1.7
ANDROGEN_RESPONSE	1.6	1.8	1.6
EPITH_MESENCHY_TRANSITION	2.3	1.2	1.5
GLYCOLYSIS	1.5	1.2	1.3
INFLAMMATORY_RESPONSE	2.1	1.1	1.3
UV_RESPONSE_DN	2.2	2.2	2.0
INTERFERON_GAMMA_RESPONSE	2.3	1.3	0.0
INTERFERON_ALPHA_RESPONSE	2.3	1.5	0.0
COMPLEMENT	2.2	1.4	1.0
IL6_JAK_STAT3_SIGNALING	2.1	1.1	1.0
KRAS_SIGNALING_UP	2.0	1.1	1.3
COAGULATION	2.0	0.0	0.0

C

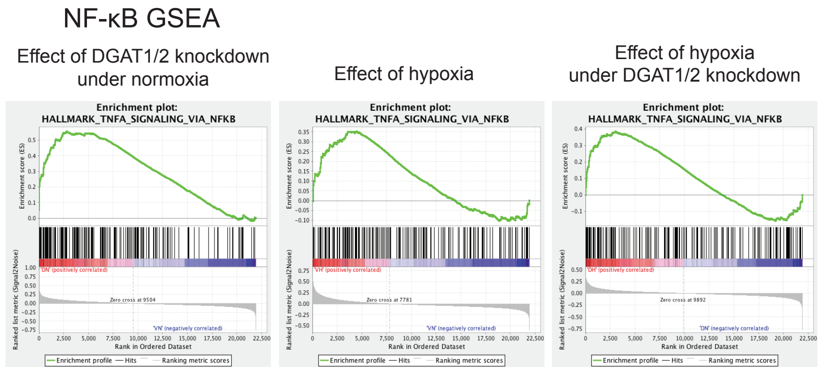

D

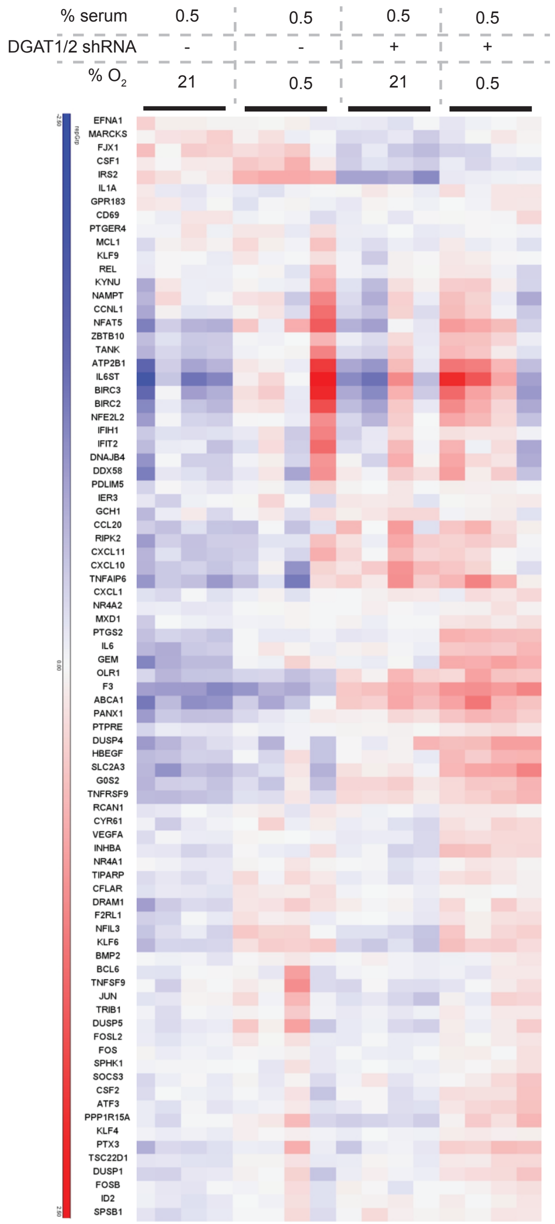

Figure S4. Related to Figure 4.
(A) Schematic of experimental workflow: DGAT1/2 shRNA A498 cells were treated with doxycycline for 5 days before being exposed to normoxia or hypoxia under serum deprivation for 48h. Samples were then harvested for total RNA and microarray analysis. (B) Normalized enrichment score from GSEA on microarrays described in (A). (C) NF-κB signature displayed for three separate comparisons. (D) Heatmap depicting levels of expression of genes composing the "leading edge" of the NF-κB signature. All microarrays were performed using 4 replicates/condition.

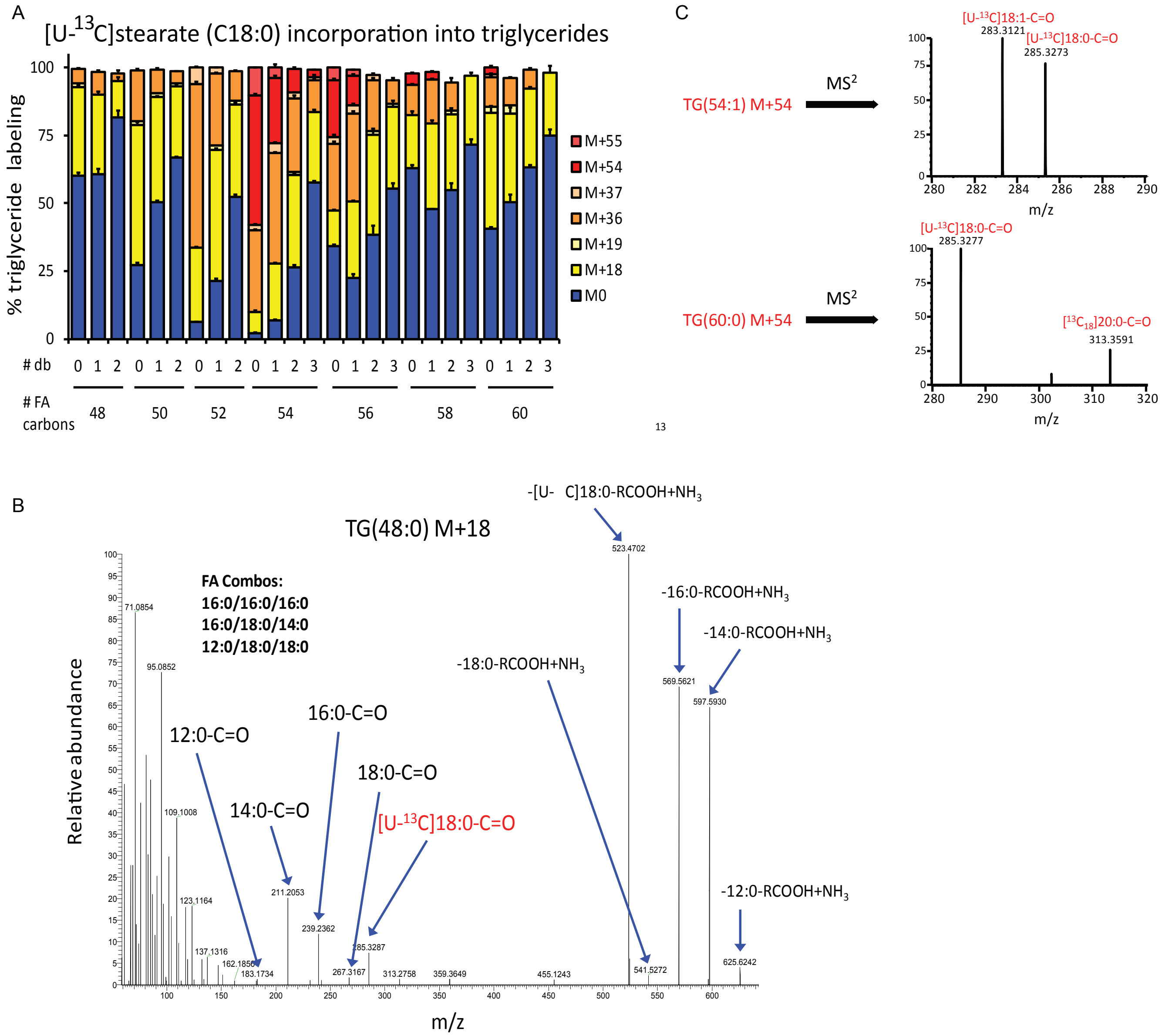

Figure S5. Related to Figure 3 and STAR Methods
(A) Observed TG labeling patters in A498 cells following 6h incubation with 25 μM [U13C]-stearate (C18:0). (B) Fragmentation pattern (MS2) of TG(48:0) containing one 13C-18 FA (M+18). (C) Evidence of elongation and desaturation of [U13C]-18:0 by cells, by assessment of acylium ions of FAs in MS2.
